# Latent Autoimmune Diabetes in a Young Female: A Case Report

**DOI:** 10.7759/cureus.70202

**Published:** 2024-09-25

**Authors:** Keerthana K Pampapathi, Hsu Y Mon, Joshua M Ibrahim, Samson O Oyibo

**Affiliations:** 1 Medicine, Peterborough City Hospital, Peterborough, GBR; 2 Diabetes and Endocrinology, Peterborough City Hospital, Peterborough, GBR

**Keywords:** diabetes mellitus, diabetes mellitus type 1.5, beta cell function, young people, latent autoimmune diabetes

## Abstract

Latent autoimmune diabetes (LAD) involves gradual autoimmune destruction of the pancreatic beta cells, leading to reduced insulin production. When it occurs in adults, the term “latent autoimmune diabetes in adults (LADA)” is used, and when it occurs in young people, the term “latent autoimmune diabetes in the young (LADY)” is used. Patients usually present with symptoms suggestive of type 2 diabetes, and test positive for islet cell antibodies, but do not require insulin therapy at diagnosis and for up to six months thereafter. We report an 18-year-old female who presented with symptoms of type 2 diabetes with non-ketotic hyperglycemia, had positive testing for multiple islet cell autoantibodies, but did not require insulin therapy at diagnosis. She was initially thought to have either type 2 diabetes or maturity-onset diabetes of the young, and so treated with a sulfonylurea with good results before the addition of metformin and subsequent conversion to basal-bolus insulin therapy because of raised postprandial blood glucose levels. This case highlights the importance of improved knowledge required to prevent the misdiagnosis of LAD as type 2 diabetes, the importance of regular follow-up for these patients, and the need for a low threshold for commencing insulin therapy to prevent diabetic ketoacidosis and long-term diabetes complications in patients with LAD.

## Introduction

Latent autoimmune diabetes in adults (LADA), also called slow-onset type 1 diabetes or type 1.5 diabetes, involves gradual autoimmune destruction of beta cells, leading to reduced insulin production [1]. It is distinct from type 1 diabetes, in which insulin therapy is required from diagnosis, and from type 2 diabetes, in which insulin therapy is not required at all, or at least not until several years after diagnosis. Patients usually present over the age of 30 years with symptoms suggestive of type 2 diabetes and test positive for islet cell antibodies, but do not require insulin therapy at diagnosis and for up to six months thereafter [1]. LADA can also occur in young people, where it is called latent autoimmune diabetes in the young (LADY). They present in the same way, that is, with symptoms of type 2 diabetes, test positive for islet cell autoantibodies, and again do not require insulin therapy at diagnosis and for six months afterward [2].

The occurrence of latent autoimmune diabetes (LAD) in childhood and adolescence can pose a diagnostic challenge to healthcare workers, especially in primary care, where the patients first present. There is no established definition or diagnostic criteria for LADY. An adolescent presenting with a few months’ duration of polyuria, polydipsia, and weight loss with non-ketotic hyperglycemia and not requiring insulin therapy at diagnosis could be misdiagnosed as having type 2 diabetes or maturity-onset diabetes of the young (MODY). We report an 18-year-old female who presented with symptoms of type 2 diabetes with non-ketotic hyperglycemia, and tested positive for multiple islet cell autoantibodies but did not require insulin therapy at diagnosis.

## Case presentation

Medical history and demographics

An 18-year-old female was seen in the adolescent diabetes clinic, having been recently diagnosed with diabetes not requiring insulin therapy. She had originally presented to her general practice with symptoms of polyuria, polydipsia, and weight loss that had been ongoing for three months. Her general practice thought she may have type 2 diabetes or MODY. She had no significant past medical history. She had been started on gliclazide 40 mg twice a day. She was not on any other medication. She had no family history of diabetes. On examination, she was clinically stable and had no clinical features of Cushing's syndrome, acromegaly, or hyperthyroidism. She weighed 68 kg with a height of 1.58 m (body mass index: 27.4 kg/m²). 

Investigations

At initial presentation to her general practitioner, her blood glucose was 23 mmol/L with normal capillary and urine ketone levels. A glycated hemoglobin level of 92 mmol/mol indicated several weeks of hyperglycemia. Her full blood count, liver, renal, and thyroid function were normal. Her C-peptide level was in the normal range, indicating clinically significant insulin production. She had a very high titer of glutamic acid decarboxylase autoantibodies (GADA), in keeping with a diagnosis of autoimmune diabetes (Table 1).

**Table 1:** Results of investigations at initial presentation to general practice Results confirm the existence of non-ketotic hyperglycemia for the past three months. GADA: glutamic acid decarboxylase autoantibodies; ICA: islet cell cytoplasmic autoantibodies

Parameter tested	Results	Laboratory reference ranges
Hemoglobin (g/L)	138	130-180
White cell count (10^9^/L)	8.4	4.0-11.0
Platelet count (10^9^/L)	405	150-400
Sodium (mmol/L)	135	133-146
Potassium (mmol/L)	4.5	3.5-5.3
Chloride (mmol/L)	100	59-104
Creatinine (µmol/L)	56	45-84
Glucose (mmol/L)	23	4.0-7.0
Thyroid-stimulating hormone (mU/L)	1.14	0.3-4.2
Glycated hemoglobin (mmol/mol)	92	<42
Albumin (g/L)	46	35-50
Alanine transferase (U/L)	11	10-60
Alkaline phosphatase (U/L)	76	30-130
Total bilirubin (µmol/L)	8	<21
Capillary ketones (mmol/L)	0.2	<0.4
C-peptide (nmol/L)	0.99	0.37-1.47
GADA (IU/mL)	>2000	<10
Non-specific ICA	Positive	Negative
Urine ketones (mmol/L)	<0.5	<0.5

Treatment

The patient was initially started on gliclazide in primary care. A repeat glycated hemoglobin assessment at the adolescent clinic demonstrated that her glycated hemoglobin level had improved to 50 mmol/mol three months after commencing the gliclazide. Considering this, she wanted to continue the gliclazide despite discussions about the risk of possible gliclazide-induced beta-cell exhaustion. Other test results revealed a positive test for tyrosine phosphatase-related islet antigen-2 autoantibodies (IA-2A), zinc transporter 8 autoantibodies (ZnT8A), and non-specific islet cell cytoplasmic autoantibodies (ICA), all in keeping with autoimmune diabetes (LAD) not requiring insulin therapy (Table 2).

**Table 2:** Results of investigations at the adolescent clinic three months after the initial presentation The patient is demonstrating multiple beta-cell autoantibodies positive while not requiring insulin therapy. GADA: glutamic acid decarboxylase autoantibodies; IA-2A: islet antigen-2 autoantibodies; ZnT8A: zinc transporter 8 autoantibodies

Blood test	Results	Laboratory reference ranges
Glycated hemoglobin (mmol/mol)	50	<42
C-peptide (nmol/L)	1.07	0.37-1.47
GADA (IU/mL)	>2000	<10
IA-2A (U/mL)	>4000	<10
ZnT8A (U/mL)	>2000	<15

Outcome and follow-up

Six months after commencing gliclazide, her postprandial blood glucose levels rose to 10-20 mmol/L, and her glycated hemoglobin level rose to 75 mmol/mol. We discussed conversion to insulin, but she was against this and wanted the addition of metformin. However, a month later, she was converted to basal-bolus insulin therapy during a visit to the emergency department with symptoms of dizziness and dehydration due to hyperglycemia. Her serum ketones and blood pH were normal at that time. Three months after conversion to basal-bolus insulin therapy, her glycated hemoglobin level improved to 61 mmol/mol. There was further improvement in her glycated hemoglobin level to 51 mmol/mol three months later. Her C-peptide levels and serum ketone levels remained in the normal range throughout (Table 3). The patient continued regular follow-ups with the adolescent diabetes clinic.

**Table 3:** Results of investigations at six, nine, and 12 months after the initial presentation Insulin therapy started six months after presentation (a total of nine months after symptoms began).

Blood test	Results at six months	Results at nine months	Results at 12 months	Laboratory reference ranges
Glycated hemoglobin (mmol/mol)	75	61	51	<42
C-peptide (nmol/L)	1.03	-	-	0.37-1.47
Capillary ketones (mmol/L)	0.2	-	-	<0.4

## Discussion

We have reported a young female who presented with features of type 2 diabetes at her primary care center. Because of her presentation, she was suspected to have type 2 diabetes or MODY and was commenced on a sulfonylurea (gliclazide) with good improvement in symptoms and glucose levels. Subsequent pancreatic autoimmune antibody analysis demonstrated the presence of multiple islet cell antibodies, which indicated a diagnosis of LAD. The patient wanted to continue gliclazide despite the risk of possible gliclazide-induced beta-cell exhaustion, with the subsequent addition of metformin. However, the patient was eventually converted to basal-bolus insulin therapy because of uncontrolled hyperglycemia.

MODY is a form of monogenic diabetes with an autosomal dominant inheritance pattern. Patients have a strong family history of diabetes, typically presenting in the second to fifth decade of life. Patients present with features of both type 1 and type 2 diabetes. They are lean, non-ketotic, usually under the age of 25 years, and do not test positive for pancreatic autoantibodies. They have beta-cell dysfunction but still have preserved insulin production, as measured by raised C-peptide levels. Sulfonylureas can be used as an initial treatment. Genetic testing is required in line with diagnostic guidelines [3,4]. Our patient had a similar presentation but tested positive for multiple pancreatic autoantibodies, had normal C-peptide levels, and did not have a family history of diabetes.

LAD is due to the gradual autoimmune destruction of the pancreatic beta cells, leading to a gradual decline in insulin production [1,2]. This can occur in adults (LADA) and in children (LADY). Patients present with polyuria, polydipsia, fatigue, visual changes, and weight loss. Diabetic ketoacidosis is rarely the first presentation of LADA. Patients usually test positive for pancreatic autoantibodies, namely GADA, plus/minus tyrosine phosphatase-related IA-2A, insulin autoantibodies (IAA), ZnT8A, and non-specific ICA. Patients do not require insulin therapy for at least six months from diagnosis. Diet-alone and/or oral anti-diabetic medication is the usual treatment regimen. Sulfonylureas are avoided because they may speed up beta-cell fatigue [1,2]. However, studies have demonstrated that early use of insulin therapy may preserve beta-cell function in these patients [5]. Our patient had a similar presentation and did not require insulin therapy at diagnosis or for more than six months of follow-up. These features were in keeping with LADY.

The diagnostic criteria for LADA include adult age of onset (>30 years), the presence of any islet cell autoantibody, and the absence of insulin requirement for at least six months from diagnosis [6]. However, the first and third criteria remain controversial because the use of insulin therapy at presentation, especially during an acute admission to the hospital and for a short time thereafter, is physician-dependent, and LAD can occur at any age [7].

The term “latent autoimmune diabetes” has always been associated with adults, so the literature concerning young people under 30 years old is sparse. Only a few case reports exist [8,9]. In patients above the age of 30, about 2.6%-11.7% of individuals with phenotypic type 2 diabetes at diagnosis have evidence of β-cell autoimmunity and are therefore diagnosed as having LADAs [5]. These autoimmune-positive individuals are more likely to become insulin-dependent earlier than individuals with autoantibody-negative type 2 diabetes. A recent study demonstrated a high prevalence of LADY (9%) in youth with a type 2 diabetes phenotype. The authors concluded that LAD forms a continuous age-related spectrum from LADY to LADA, in which LADY shows greater autoimmunity [10]. The study group also acknowledged the lack of established definitions and diagnostic criteria for LADY [10].

There have been promising results with trials of immunotherapies for LAD and type 1 diabetes. However, the beta-cell damage is often severe, such that immunotherapies alone cannot reverse the damage. Research into slowing beta-cell loss and extending the duration of glycemic control is ongoing [11].

There should be a low threshold for starting insulin therapy for young people with LAD exhibiting evidence of fasting or postprandial hyperglycemia with rising glycated hemoglobin levels while on a trial of diet control or oral anti-diabetic therapy. Some patients may require insulin therapy at initial presentation during an acute admission.

## Conclusions

LAD can occur at any age, leading to the terms LADA and LADY. Young patients with a type 2 diabetes phenotype should have their pancreatic islet cell autoantibodies assessed. After a diagnosis of LAD at any age, patients require regular follow-up with glucose, glycated hemoglobin, and C-peptide measurements to assess the need for insulin therapy to prevent diabetic ketoacidosis and long-term diabetes complications. Since LAD can occur at any age, we propose that the age criterion be removed, and the terms LADA and LADY be replaced by the single term “latent autoimmune diabetes (LAD)”.
